# Dose-Related Urinary Metabolic Alterations of a Combination of Quercetin and Resveratrol-Treated High-Fat Diet Fed Rats

**DOI:** 10.3389/fphar.2021.655563

**Published:** 2021-04-16

**Authors:** Tongxi Zhuang, Xinhua Liu, Wen Wang, Jing Song, Le Zhao, Lili Ding, Li Yang, Mingmei Zhou

**Affiliations:** ^1^ Institute for Interdisciplinary Medicine Sciences, Shanghai University of Traditional Chinese Medicine, Shanghai, China; ^2^ Experiment Center for Science and Technology, Shanghai University of Traditional Chinese Medicine, Shanghai, China; ^3^ School of Rehabilitation Science, Shanghai University of Traditional Chinese Medicine, Shanghai, China; ^4^ The Ministry of Education (MOE) Key Laboratory for Standardization of Chinese Medicines and The State Administration of Traditional Chinese Medicine (SATCM) Key Laboratory for New Resources and Quality Evaluation of Chinese Medicines, Institute of Chinese Materia Medica, Shanghai University of Traditional Chinese Medicine, Shanghai, China

**Keywords:** resveratrol, quercetin, high-fat diet, obesity, gas chromatography-mass spectrometry, metabolomics

## Abstract

Most herbal polyphenols and flavonoids reveals multiple ameliorative benefits for obesity caused by chronic metabolic disorders. Accumulated studies have revealed that preferable therapeutic effects can be obtained through clinical combination of these two kinds of natural compounds for obesity improvement. The typical representative research was the combination of quercetin and resveratrol (CQR), in which the ratio of quercetin and resveratrol is 2:1, demonstrating a synergistic effect in anti-obesity process. Although there exists reports clarifying the mechanism of the combination of two to improve obesity from the perspective of improving adipose tissue inflammation or modulating the composition of intestinal flora, there are few further studies on the mechanism of drug action from the perspective of metabolites transformation. In this research, we mainly focused on the alterations of endogenous metabolites in rats, and analyzed the urine metabolites of obese and intervention model. Therefore, a gas chromatography-mass spectrometry (GC-MS) based metabolomics approach was applied to assess the potential effects and mechanisms of CQR at different dosages (45, 90, and 180 mg/kg) in high fat diet (HFD)-induced obesity rats. Body weight gain and visceral fat weight were reduced by CQR, as well as blood lipid and inflammatory factor levels were increased by CQR in a dose-related manner. Urinary metabolomics revealed 22 differential metabolites related to the HFD-induced obesity, which were reversed in a dose-dependent manner by CQR, of which 8 were reversed in the 45 mg/kg CQR group, 15 were reversed in the 90 mg/kg CQR group, and 18 were reversed in the 180 mg/kg CQR group. Combined with bioinformatics and pattern recognition, the results demonstrated that the key differential metabolites were basically involved in amino acid metabolism, galactose metabolism, pantothenate and CoA biosynthesis, pyruvate metabolism and lysine degradation. In summary, our results showed significant therapeutic action by CQR administration and remarkable metabolomic changes after HFD feeding and CQR intervention. Urinary metabolomic analysis was highlighted on account of providing holistic and comprehensive insights into the pathophysiological mechanisms of the HFD-induced obesity, which also supplied clues for the future mechanism studies of CQR’s anti-obesity effects.

## Introduction

Polyphenols, derived from diet food such as vegetables and fruits, as well as beverages such as juice, coffee and tea, are one of the most interested goals of researchers among the phytochemicals studied for the treatment of obesity (Farhat et al., 2017). Quercetin (3,3′,4′,5,7-pentahydroxyflavone), and resveratrol (3,5,4′-trihydroxytrans-stilbene), both are polyphenolic nutraceuticals belonging to the flavonoid category of natural compounds, possess a variety of health beneficial properties, especially their antioxidant effect (Gelen et al., 2017). Each of them is among the most frequently reported natural polyphenols with anti-obesity effects in animal and *in vitro* studies, as well as in humans (Arias et al., 2016; Zhao Y. et al., 2017). Furthermore, the combination of the two emerged a synergistic effect in anti-obesity studies, which are responsible for activating the AMPK pathway and inhibiting adipocytes inflammatory responses (IL-2, IL-6, IFN-γ, and TNF-α, etc.) (Zhao et al., 2017a). It is worth mentioning that the CQR has been widely employed in the treatment of metabolic diseases, and as a therapeutic regimen of natural products, its clinical application has been promising and preferable for conjoint efficacy with few report on its adverse reaction. In previous research, we also found that CQR in a ratio of 2:1 showed synergistic effects in high fat diet (HFD) -fed mice at both transcriptional and metabolic levels (Zhou et al., 2012). CQR in the same ratio showed efficacy on the dysbacteriosis in HFD-fed rats.

Metabolomics generally targets at all the low molecular weight metabolites of a certain organism or cell in a specific physiological period by qualitative and quantitative assessment at the same time. It is a branch of systems biology based on cluster index analysis, high throughput detection and data processing, and aiming at information modeling and system integration (Klassen et al., 2017; Jiang et al., 2019). Metabolomics emphasizes on small molecule metabolites as substrates and products of various metabolic pathways, which has been extensively used to explore disease pathogenesis, evaluate treatment effects and identify disease biomarkers (Puchades-Carrasco and Pineda-Lucena, 2015). Gas chromatography-mass spectrometry (GC-MS), one of the main metabolomic technology platforms, featured by the advantages of high resolution, high sensitivity, comparatively standard databases (NIST, Willey and other complete databases), easy to qualitative analysis, has been widely applyed in metabolomic research. Urine, as the final product of human metabolic network, contains more metabolites than other substrates, which is favorable for providing hints for the discovery of new biomarkers from the perspective of metabolism. In addition, as an analytical sample, it is non-invasive and easy to obtain, and has become the main research object of metabolomics.

In recent decades, with the improvement of the quality of social living standards, the high mortality caused by obesity, has evolved into an invisible killer of human health issue due to long-term unbalanced calorie intake and metabolic consumption. From the essence of pathology, obesity is characterized by system low-grade inflammation and excessive adipose tissue accumulation induced by individual metabolic disorder. As to targeting at the crucial differential biomarkers in metabolic organism, it will provide instructive clinical guidance for the improvement of obesity.

In this study, based on a urinary GC-MS metabolomic approach, the dose-related alterations of CQR treated HFD fed rats were investigated. In addition, the relevant differential metabolites and metabolic pathways were further identified.

## Experimental Procedures

### Animal Model

Male Wistar rats, aged 10 weeks, weighted 160–180 g, were obtained from Shanghai SLAC laboratory Animal Co. Ltd. (Shanghai, China), and then housed in a single cage separately in temperature controlled (22 ± 0.5°C), relative humidity-controlled (40–70%) and light-controlled (12 h/12 h light-dark cycle) animal facility, with free access to standard chow and water. After a 7-days adaptation, rats were randomly divided into the following two groups: normal diet group (N, *n* = 10) and HFD group, and their formulas was same as our previous study (Zhao et al., 2018). The obesity-resistant rats with lower body weight gain were picked out after 2 weeks of HFD treatment (Li et al., 2008). The rest of rats in HFD group were randomly divided into model group (M, *n* = 10) and CQR treatment groups (*n* = 30) and continued to be fed with HFD.

The mixture of resveratrol (R; 98% purity; 3,4′,5-trihydroxystilbene; Hangzhou Ruishu Biochemical Co., Ltd.; 501-36-0) and quercetin (Q; 95% purity; 3,3′,4′,5,7-pentahydroxyflavone; Nanjing ZeLang Biological Technology Co., Ltd.; 117-39-5) was dissolved in 0.5% carboxymethylcellulose sodium (CMC-Na) solution and rats of CQR treatment groups were randomly divided into three groups by administered different dose of CQR mixture with a fixed composition ratio (Q:R = 2:1): high dose group (HD; *n* = 10; Q: 120 mg/kg; R: 60 mg/kg; i.g.), middle dose group (MD; *n* = 10; Q: 60 mg/kg; R: 30 mg/kg; i.g.) and low dose group (LD; *n* = 10; Q: 30 mg/kg; R: 15 mg/kg; i.g.). Rats of normal group and model group were given 0.5% CMC-Na solution (1 ml/100 g; i.g.).

### Sample Collection and Preparation

Animals were dosed daily between 9 and 10 a.m. for 8 weeks. The body weight and food intake of each rat were recorded weekly. Metabolic cages were used for collection of 24-h urine sample of each rat at the end of 10-week period experiment. The urine samples were collected and centrifuged (1,500 × g, 5 min) at 4°C. The supernatant was removed and stored at −80°C until metabolic analysis. The rats were anesthetized and blood was collected from the abdominal aorta. The blood samples were centrifuged at the force of 3,500 × g for 15 min at 4°C to separate the serum for biochemical parameters analysis. The epididymal adipose tissues (EATs), perinephric adipose tissues (PATs), and subcutaneous adipose tissues (SATs) were dissected and weighed. In addition, EATs sections were fixed in 4% formalin solution for histological analysis. All experimental procedures in this study were performed according to the Guidelines for Animal Experimentation of Shanghai University of Traditional Chinese Medicine.

### Serum Parameters Analysis

Serum lipid parameters such as triglyceride (TG), total cholesterol (TC), high-density lipoprotein cholesterol (HDL-C) and low-density lipoprotein-cholesterol (LDL-C) were measured on a Hitachi 7,600 automatic biochemical analyzer (Hitachi, Ltd., Tokyo, Japan) with the corresponding commercial kits. Moreover, according to the manufacturer’s protocols, the concentrations of serum insulin, adiponectin, leptin, and pro-inflammatory cytokines were quantified using the corresponding commercial kits (Cusabio, Barksdale, Delaware, United States).

### Histological Analysis of Epididymal Adipose Tissues

EATs were fresh isolated from rats of five groups, and further collected and fixed in 4% formalin for 24 h, then dehydrated, and embedded in paraffin. Finally, they were sectioned at a thickness of 5 μm. To determine the size of the adipose tissue, a portion of the EATs sections were stained with hematoxylin and eosin for 30 min. Five fields from each section were randomly examined using an optical microscope (Olympus BX51) and adipocyte size was measured using Image-Pro Plus 6.0 (Media Cyberetics).

### Urinary Metabolic Profiling

As described in our previous article (Zhao et al., 2018), urine samples of 1.0 ml were centrifuged at 12,000 rpm, 4°C for 10 min. Supernatants of 100 μl were collected, added with 40 μl of urease (148 IU/mg) in each tube, vortexed for 30 s, and incubated at 37°C for 15 min. Then pretreated with 10 μl of myristic acid in methanol (1 mg/ml) as an internal and 800 μl of methanol, vortexed 1 min, and centrifuged at 13,000 rpm (4°C) for 10 min 200 μl of supernatant was transferred to GC vial and dried at 30°C in a stream of pure nitrogen. Then 50 μl of methoxyamine hydrochloride in pyridine (15 mg/ml) was added to the residue of each sample. After vortex for 1 min, the mixture was incubated at 30°C for 90 min. Subsequently, 30 μl of derivative reagents [N, O-bis(trimethylsilyl) trifluroacetamide with 1% trimethylchlorosilane] were added to mixture of each, vortex oscillated for 30 s and incubated at 70°C for 60 min.

According to the previous method (Hu et al., 2017; Zhao et al., 2018), Agilent 6890N gas chromatography coupled to an Agilent 5975B Mass-Spring-Damper (MSD) system (Agilent Technologies, California, United States) was used to analyze the derivatized samples. Briefly, the derivatives separation was performed on an Agilent DB-5MS column (30 m × 0.25 mm × 0.25 μm) with a splitless mode high-purity helium at a constant flow rate of 1 ml/min, was used as carrier gas, and the solvent delay time was 5.0 min. Initially, the oven temperature was maintained at 80°C for 2 min, ramped to 160°C at a rate of 2.5°C/min, then raised to 240°C at a rate of 5°C/min, and finally fixed at 240°C for 16 min. The temperature of MS source and MS quadrupole was set to 230°C and 150°C, respectively. In addition, the electron impact ionization was 70 eV and Mass data was obtained in a full scan mode (*m/z* 30-550).

### Data Processing and Statistical Analysis

The original data analyzed by GC-MS were exported in Net CDF format through Agilent MSD workstation and preprocessed with the R 2.13.2 (Lucent Technologies) in a further step. The final data set included the sample information, peak area and retention time. Then, Microsoft Excel 2016 was applied to normalize the data to the total peak area, and the resulting data was carried out the multivariate analysis with the software of SIMCA-P 11 (Umetrics, Umeå, Sweden). Unsupervized principal component analysis (PCA) was employed to describe the metabolic profiles among groups, and supervised partial least squares discriminant analysis (PLS-DA) was performed for discriminating groups. Furthermore, the differential metabolites of variable importance in the projection (VIP) value greater than 1.0 were picked from the orthogonal partial least squares discriminant analysis (OPLS-DA) model. Using Kyoto Encyclopedia of Genes and Genomes (KEGG; http://www.genome.jp/kegg/) to confirmed potential biomarkers. Then, Metabo Analyst 3.0 (http://www.metaboanalyst.ca/) was applied to identify the metabolic pathways of differential metabolites, and Cytoscape (v3.6.1) was used to make the interaction network of metabolic pathway.

Data from serum biochemical analysis were analyzed using SPSS 21.0. Statistical analysis was performed by one-way analysis of variance (ANOVA). The results were expressed as mean ± SD. Significant differences were considered when *p* < 0.05.

## Results

### Anti-Obesity Effect of CQR in HFD-Induced Obese Rats

**FIGURE 1:**
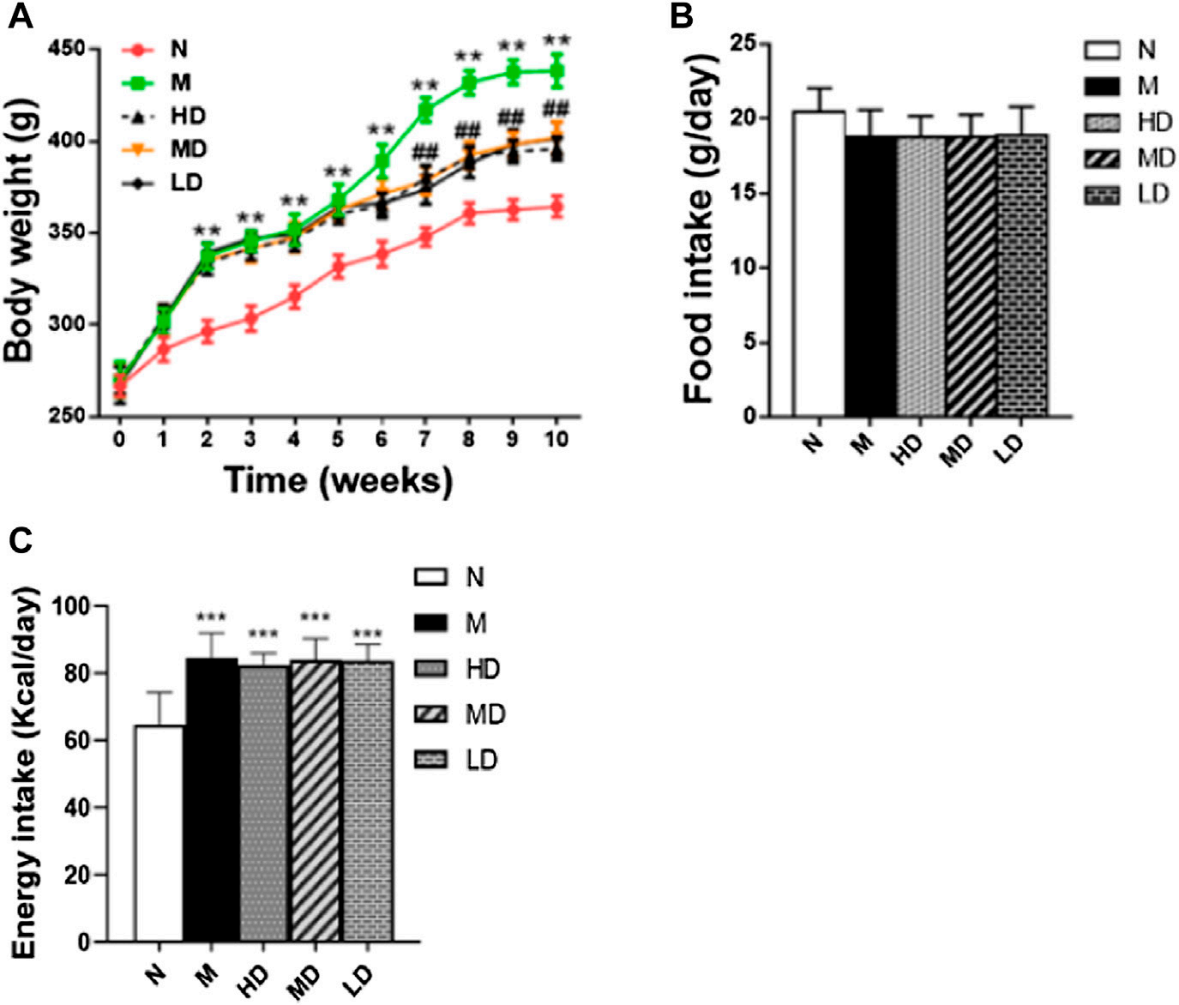
Effects of CQR on body weight. **(A)**, food intake. **(B)** and Energy intake. **(C)** in rats fed with high fat diet. Data are expressed as mean ± SEM (*n* = 10). *p < 0.05, **p < 0.01, ***p < 0.001 *vs.* N group; #*p* < 0.05, ##*p* < 0.01 *vs.* M group (N, normal diet group; M, model group; HD, high dose group; MD, middle dose group and LD, low dose group).

After 2 weeks’ HFD feeding, the body weight of rats in M group was significantly higher than that in N group as expected (Figure 1A; *p* < 0.01), which indicated that the model of obesity was successful. However, from the 7th week to the 10th week, the body weight gain of rats in CQR treatment groups were significantly decreased compared with the M group (Figure 1A; *p* < 0.01), indicating that different concentrations CQR mixture had a certain weight-loss effect. The average food intake in each group was record from the third weeks’ HFD and CQR feeding to 10th weeks’ feeding. Moreover, there was no significant difference in food intake among five groups (Figure 1B), suggesting that CQR might not exert anti-obesity effects by reducing food intake. The results of daily calorie intake of rats showed that the energy intake of N group was significantly lower than that of other groups, and there was no significant difference between group M and multiple doses of CQR groups (Figure 1C; *p* < 0.001).

### Effects of CQR on Serum Biochemical Parameters in Obese Rats

In our study, four common biomarkers of obesity, TC, TG, HDL-C and LDL-C in serum, were tested and the results were shown in Table 1. Serum concentrations of TC (*p* < 0.05), TG (*p* < 0.05), and LDL-C (*p* < 0.01) in M group were significantly higher than those in N group, while HDL-C (*p* < 0.05) was significantly decreased. In addition, different doses of CQR mixture significantly inhibited the increase of TC in the serum of HFD-induced obese rats as compared to the M group. However, compared with the M group, the level of serum TG (*p* < 0.05) in HD and MD groups was significantly decreased, and there was no significant difference in LD group. Concentration of LDL-C (*p* < 0.05) was significantly reduced in the HD and LD groups, but there was no significant difference in MD group. The level of HDL-C (*p* < 0.05) was significantly increased in HD group, and there was no significant difference between the other two CQR treatment groups.

**TABLE 1:** Effects of CQR on serum biochemical parameters in obese rats.

Group	TC (mmol/l)	TG (mmol/l)	HDL-C (mmol/l)	LDL-C (mmol/l)
N group	1.50 ± 0.04	0.69 ± 0.10	1.01 ± 0.025	0.28 ± 0.011
M Group	1.82 ± 0.08^a^	1.38 ± 0.17^a^	0.80 ± 0.03^a^	0.46 ± 0.03^b^
HD group	1.52 ± 0.04^c^	0.70 ± 0.09^c^	0.90 ± 0.02^a^ ^,^ ^c^	0.31 ± 0.03^c^
MD group	1.57 ± 0.07^c^	0.82 ± 0.10^c^	0.82 ± 0.04^a^	0.35 ± 0.04
LD group	1.55 ± 0.05^c^	0.97 ± 0.17	0.81 ± 0.04^a^	0.31 ± 0.03^c^

Data are expressed as mean ± SD (*n* = 10).

^a^ *p* < 0.05.

^b^ *p* < 0.01 *vs*. N group.

^c^ *p* < 0.05, *vs* M group. (N, normal diet group; M, model group; HD, high dose group; MD, middle dose group and LD, low dose group).

**FIGURE 2:**
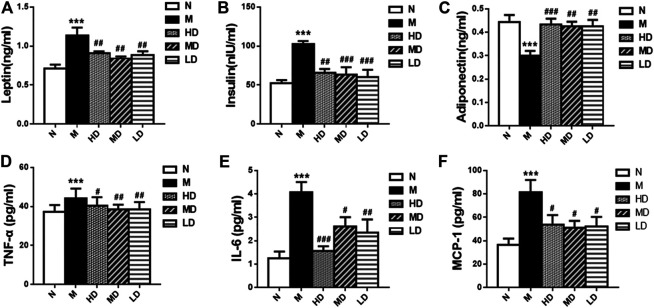
Effects of CQR on serum biochemical parameters in rats fed with high fat diet. **(A)** Leptin; **(B)** insulin; **(C)** adiponectin; **(D)** TNF-α; **(E)** IL-6; **(F)** MCP-1. Data are expressed as mean ± SEM (*n* = 10). *p < 0.05, **p < 0.01, ***p < 0.001, *vs* N group; #*p* < 0.05, ##*p* < 0.01, ###*p* < 0.001, *vs* M group.

Our results also showed levels of serum leptin, adiponectin and insulin in the five groups of rats (Figure 2). Compared with the N group, the levels of leptin (*p* < 0.001; Figure 2A) and insulin (*p* < 0.001; Figure 2B) were significantly higher in the M group, while the level of adiponectin (*p* < 0.001; Figure 2C) was significantly reduced. At the same time, different doses of CQR treatment significantly reversed the changes of leptin, adiponectin, and insulin in the M group. In addition, according to the results of ELISA, CQR inhibited the level of pro-inflammatory cytokines in serum. The levels of TNF-α (*p* < 0.001; Figure 2D), IL-6 (*p* < 0.001; Figure 2E) and MCP-1 (*p* < 0.001; Figure 2F) in the M group were increased significantly, whereas different doses of CQR inhibited the increase in the level of pro-inflammatory cytokines.

### Effect of CQR on the Morphology and Cell Diameter of Adipocytes

**FIGURE 3:**
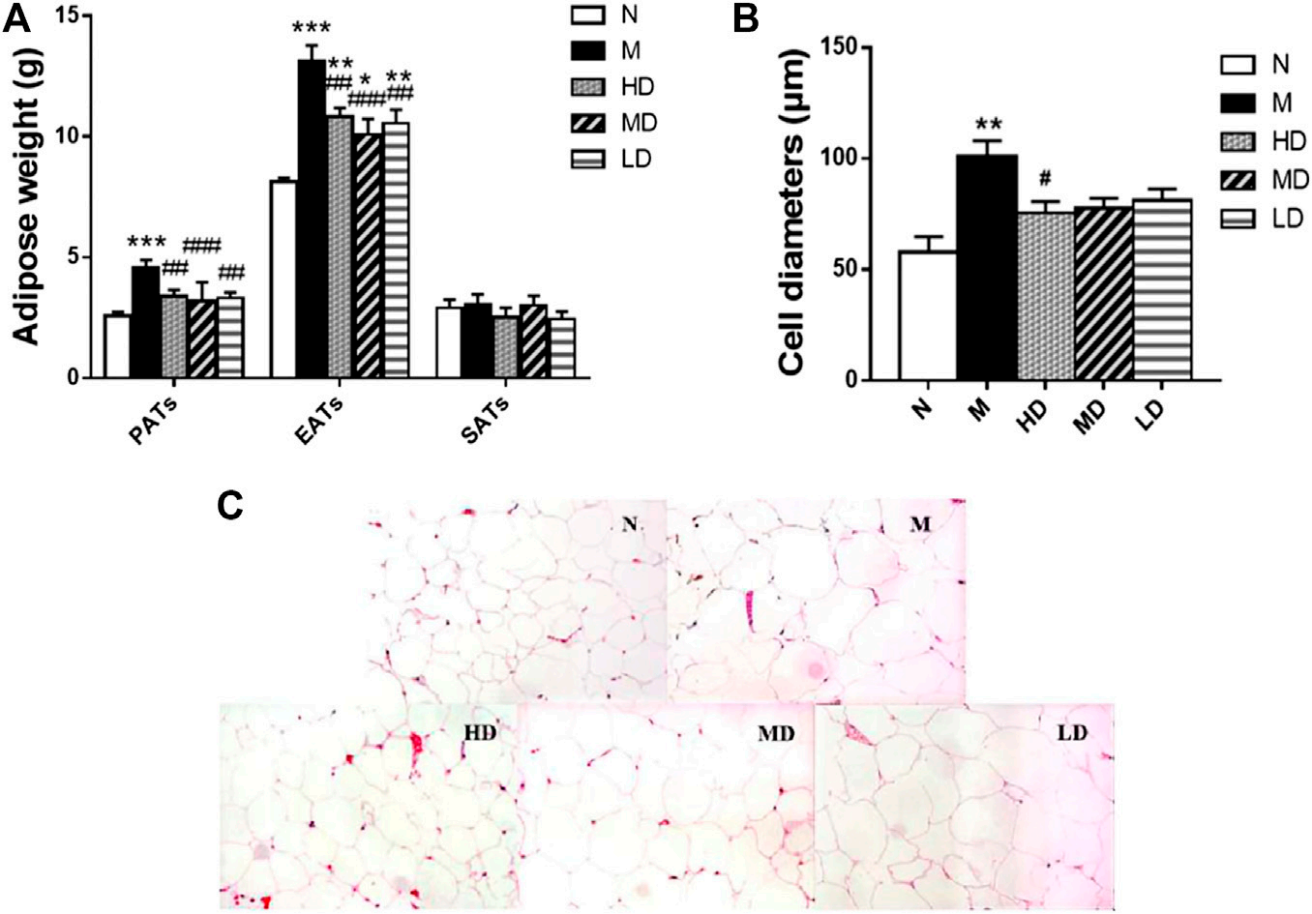
Effect of CQR on the morphology and cell diameters of adipocytes in obese rats. **(A)** The weight of perinephric adipose tissues (PATs), epididymal adipose tissues (EATs) and subcutaneous adipose tissues (SATs); **(B)** diameters of epididymal adipocyte; **(C)** epididymal adipocyte size. Data are expressed as mean ± SEM (*n* = 10). *p < 0.05, **p < 0.01, ***p < 0.001, *vs.* N group; #*p* < 0.05, ##*p* < 0.01, ###*p* < 0.001, *vs.* M group.

As shown in Figure 3, we also observed the weight of PATs (*p* < 0.001) and EATs (*p* < 0.001) in the M group was significantly higher than that in N group, and the supplement of CQR at different doses could significantly reduce the weight of the two adipose tissues, while there was no significant difference in the weight of SATs weight among five groups. In addition, the diameter of epididymal adipocyte in M group was significantly higher than that in N group (*p* < 0.01). Compared with M group, high doses of CQR significantly inhibited the increase in epididymal adipocyte diameter, while MD and LD groups had no significant difference compared with M group. These results implied that CQR could inhibit high fat diet-induced obesity.

### Urinary Metabolomic Analysis

**FIGURE 4:**
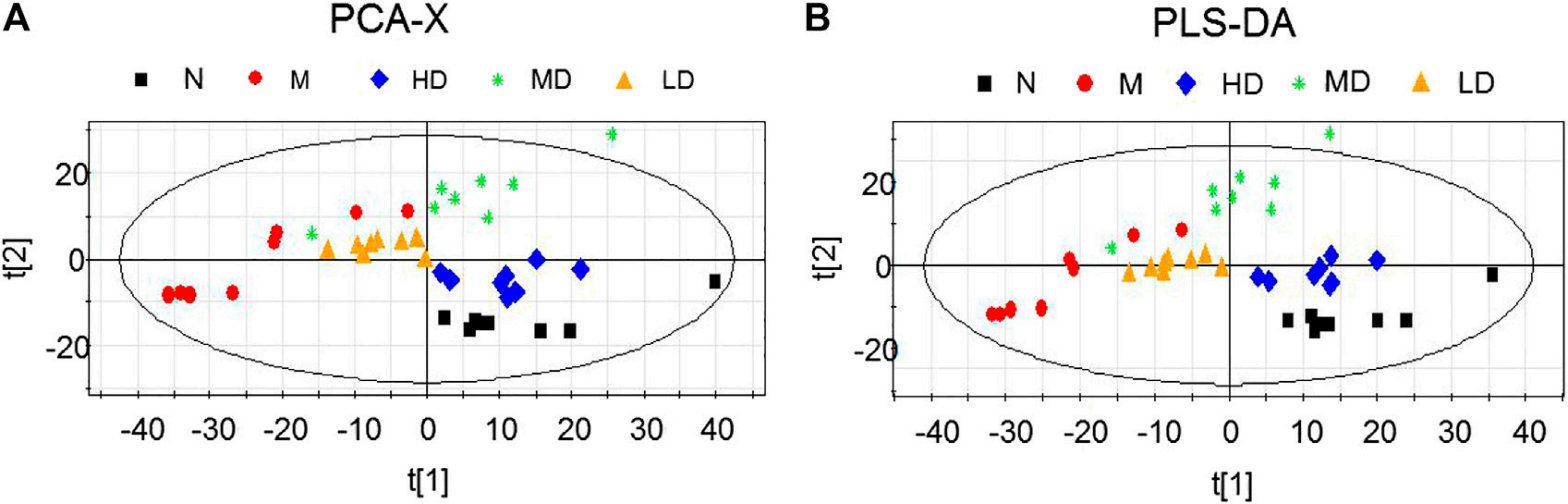
Scores plots of multivariate statistical analysis on urine samples. **(A)** Principal Component Analysis (PCA) scores plot; **(B)** Partial Least Squares-discriminant Analysis (PLS-DA) scores plot.

As the dates showed in Figure 4, 24-h urine sanples were collected and metabonomic analysis was performed The PCA score plots (*R* ^*2*^ *X* = 0.719, *Q* ^*2*^ (cum) = 0.861) and PLS-DA score plots (*R* ^*2*^ *X* = 0.768, *R* ^*2*^ *Y* = 0.975, *Q* ^*2*^ (cum) = 0.861) demonstrated significant differences between N and M group. The apparent trend of separation indicated that the metabolic level of model rats fluctuated significantly and deviated from the normal state. HD, MD, LD groups were significantly separated from M group. Additionally, the result of HD group, which was closer to the N group, laterally supported the view that the high dosage of CQR potentially displayed a superior effect on obesity treatment. The parameters of *R* ^*2*^ *X* and *Q* ^*2*^ were used to evaluate the PCA model, while *R* ^*2*^ *X*, *R* ^*2*^ *Y*, *Q* ^*2*^ (cum) were used to evaluate the PLS-DA model. *R* ^*2*^ *X*, *R* ^*2*^ *Y* were usually employed for quantifying the goodness-of-fit of model and *Q* ^*2*^ (cum) was employed to evaluate the predictability of model. The model is ideal when the value of *R* ^*2*^ *X* and *Q* ^*2*^ (cum) are close to 1. In this study, the models of PCA and PLS-DA were valid and positive.

### Differential Metabolite Analysis

**FIGURE 5:**
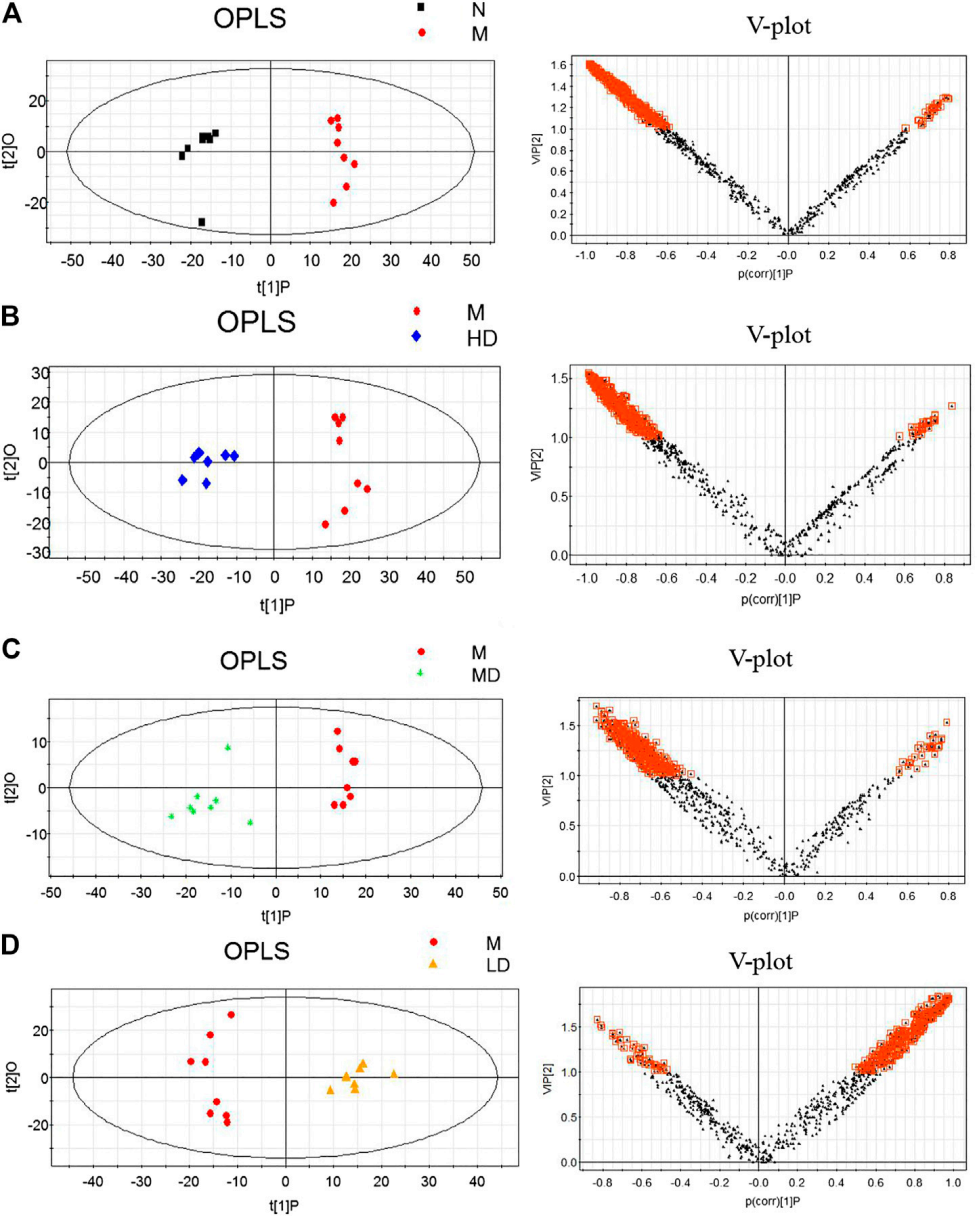
Orthogonal partial least squares-discriminant analysis (OPLS-DA) score plots (left) and corresponding loadings plots (right). **(A)** M group *vs.* N group, **(B)** HD group *vs.* M group, **(C)** MD group *vs.* M group, and **(D)** LD group *vs.* M group.

**FIGURE 6:**
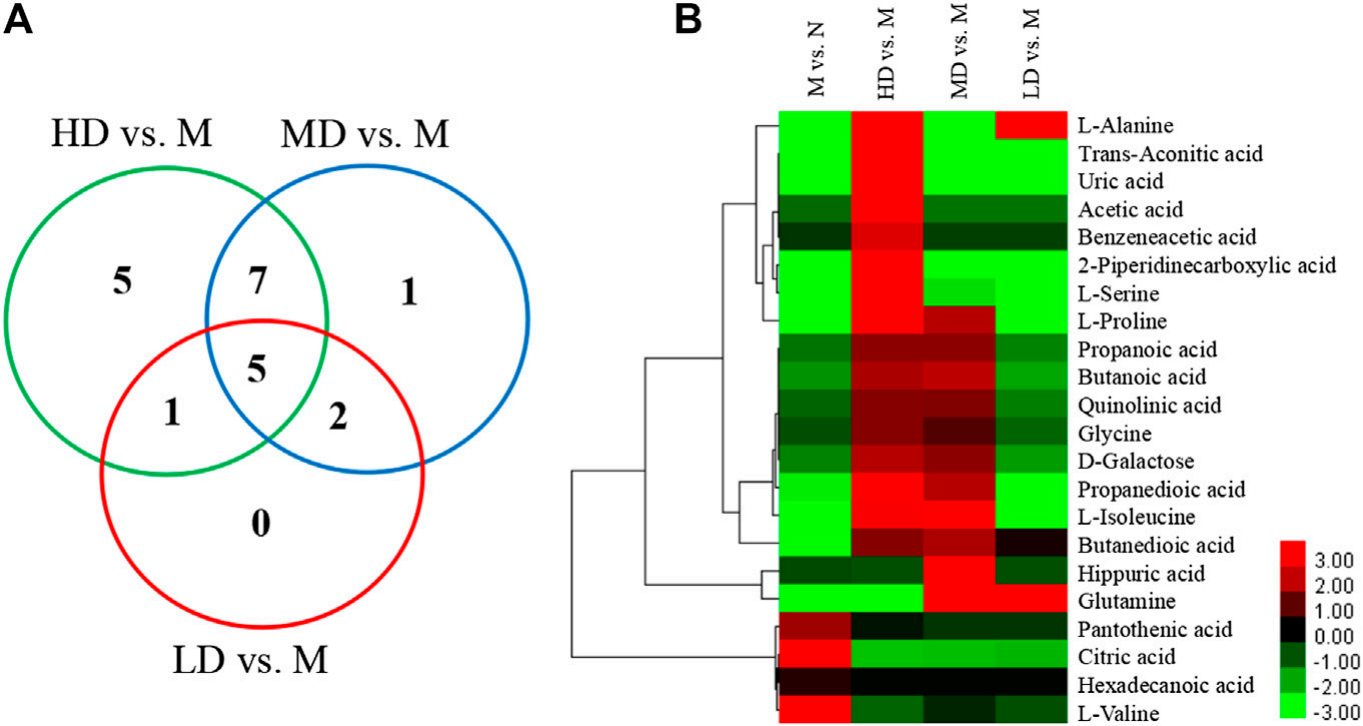
The Venn diagram and heat map of identified differential metabolites in obese rats fed a high-fat diet. **(A)** The Venn diagram presents the number of differential metabolites that were changed in the HD, MD, and LD groups compared to the M group; **(B)** the heat map presents the differential metabolites between M group vs. N group, HD group vs. M group, MD group vs. M group, and LD group vs. M group.

**FIGURE 7:**
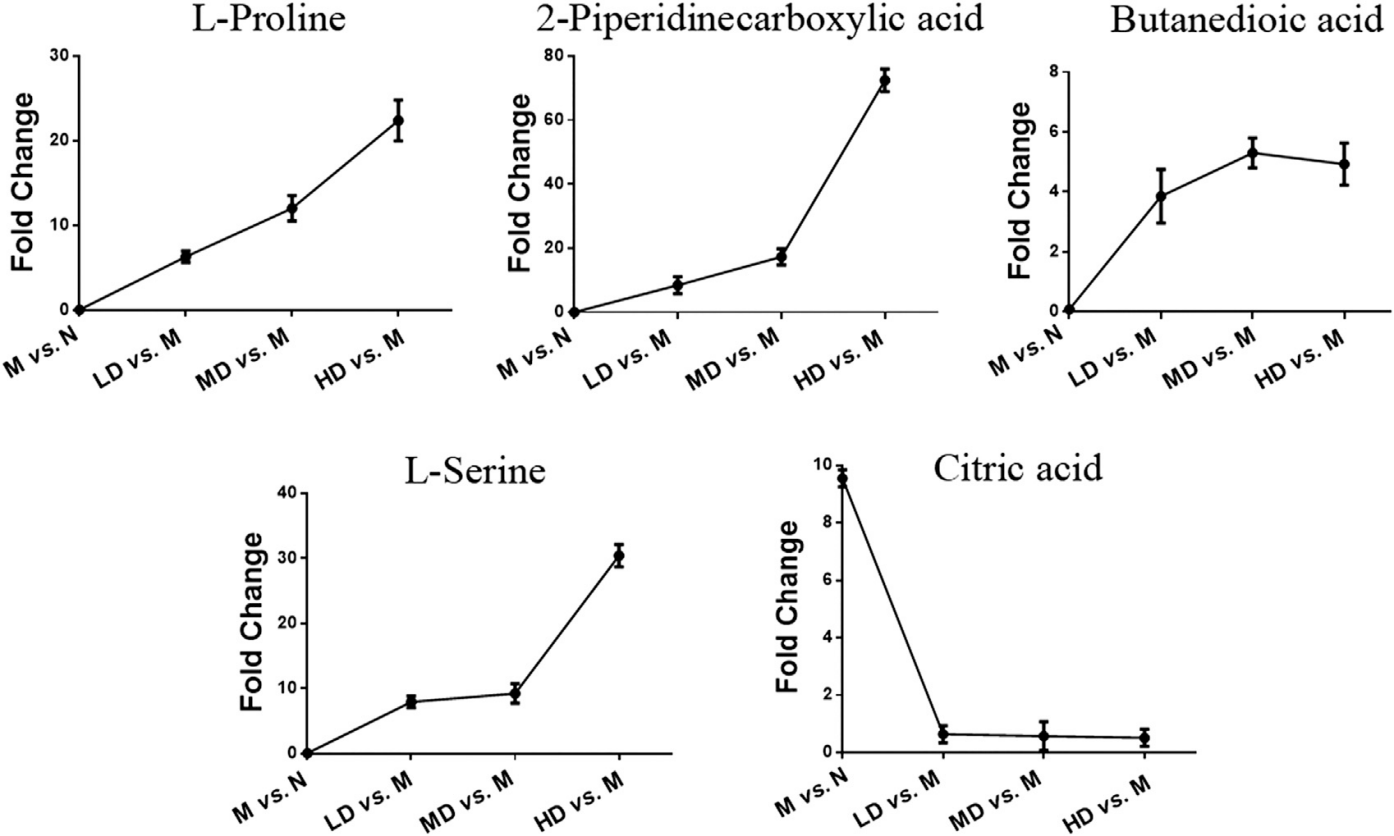
Selected differential metabolic markers. The common differential metabolites present in three of CQR-treated groups were selected as potential metabolic biomarkers.

To further understand the differential metabolites in the urine samples among groups, orthogonal partial least squares-discriminant analysis (OPLS-DA) and corresponding variable import projection (VIP) were constructed (Figure 5). In this study, metabolites with VIP >1 and paired t-test *p* < 0.05 were selected as differential metabolites. These differential metabolites were searched in KEGG database and HMDB database, and 22 different substances were identified in Supplementary Table S1. The metabolites were mainly involved in the CoA biosynthesis and citric acid cycle, as well as the intermediate products of amino acid metabolism, which proved that CQR improved the metabolism of galactose, amino acid and other nutrients. In addition, three different dose groups were more or less able to acquire weight loss through related amino acid metabolism regulation. HD group, MD group and LD group affected 18, 15, and 5 metabolites, respectively. Five of these urinary differential metabolites were common to these three groups and could be used to further analyze whether CQR produced a dose-dependent effect (Figure 6A). The effect of each dose group on differential metabolites could be visualized directly through the heat map. It can be concluded from Figure 6B that citric acid, L-Valine, and pantothenic acid were upregulated (red represents upregulation), while the remaining 19 metabolites were significantly downregulated (green represents upregulation) in M group, and each dose group selectively modulated these differential metabolites. L-Proline, 2-Piperidinecarboxylic, butanedioic acid, L-Serine and citric acid existed in all three CQR dose groups. The values of fold change were showed in Figure 7. In these five urinary differential metabolites, there were four metabolites showed dose-dependent changes, except for succinic acid. L-proline, 2-piperidinecarboxylic acid and L-serine increased in a dose-dependent manner, while citric acid decreased in a dose-dependent manner. Therefore, these changes in differential metabolites are closely related to the development of obesity, and might be potential biomarkers for obesity induced by high-fat diets. CQR could selectively regulate these metabolites to exert anti-obesity.

### Metabolic Pathway Analysis

**FIGURE 8:**
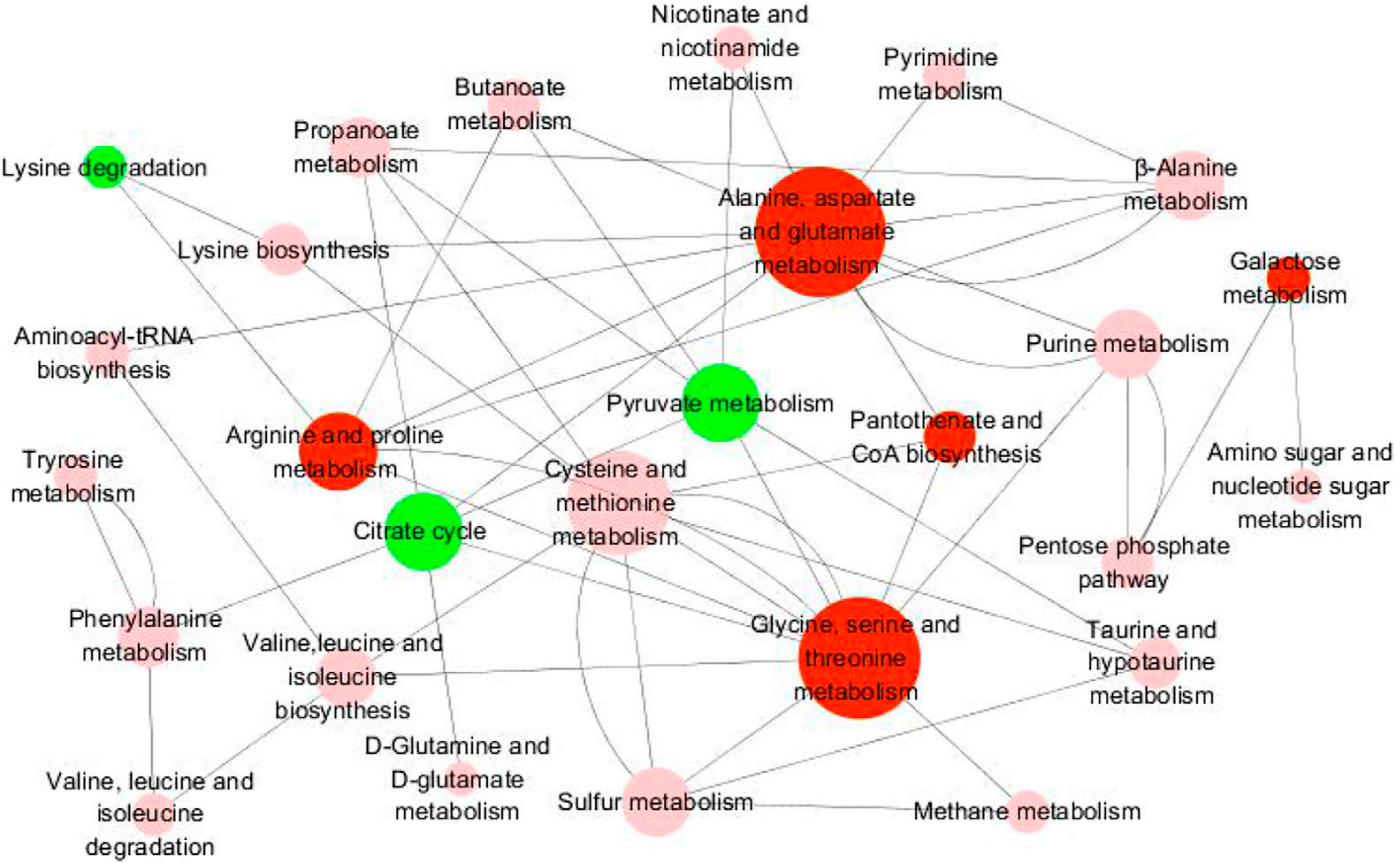
Diagram of metabolic pathway interaction network. The pathway analysis was performed with Kyoto Encyclopedia of Genes and Genomes (KEGG) database (http://www.kegg.jp/kegg) and the picture was generated by Cytoscape. The bigger node represents more pathways connection. Red nodes represent pathway impact factors greater than 0.1 (Pathway impact >0.1), green nodes represent pathway impact factors greater than 0.05 (Pathway impact >0.05), and pink nodes represent pathway impact factors less than 0.05 (Pathway impact <0.05).

Metabo Analyst 3.0 and KEGG were used to identify the disturbed metabolic pathways. Figure 8 showed the interaction network diagrams of these disturbed metabolic pathways through Cytoscape software. Alanine, aspartate, and glutamate metabolism was the metabolic pathway related to the most of other metabolic pathways, followed by glycine, serine and threonine metabolism, cysteine and methionine metabolism, arginine and proline metabolism, pyruvate metabolism, and citrate cycle. Altogether, the influence factors of 5 metabolic pathways were greater than 0.1, i.e., glycine, serine and threonine metabolism, alanine, aspartate and glutamate metabolism, galactose metabolism, pantothenate and CoA biosynthesis, and arginine and proline metabolism. In addition to the metabolic pathways described above, pyruvate metabolism, lysine degradation, and the citric acid cycle were the metabolic pathways with an influence factor greater than 0.05 (Figure 8). The metabolic pathways affecting factors greater than 0.05 and the main metabolites affecting these metabolic pathways were shown in Supplementary Table S2.

## Discussion

Recently, accumulated laboratory studies indicated that the effect of anti-obesity and amelioration of other metabolic diseases potentially were connected with combined application of polyphenols, which could exert their additive or synergistic effects through inhibiting chronic inflammation and oxidative stress (Dahiya et al., 2017; Tunapong et al., 2018). CQR, the combination of resveratrol and quercetin, exhibited a variety of pharmacological activities such as antibacterial, anti-tumor, and anti-oxidant effects, and displayed good preventive and therapeutic effects on obesity and its complications (Etxeberria et al., 2015; Zhao et al., 2017a; Yang and Kang, 2018). In previous research (Zhao et al., 2017b), we have confirmed that intake of CQR could reduce obesity in HFD fed rats through modification of gut microbiota. In this study, aiming to evaluate CQR’s systemic mechanism on anti-obesity phenotype, we observed the intervention of different doses of CQR on HFD-induced obesity rats, especially the metabolic changes. The results clearly showed that the obesity model induced by HFD was successfully established. After the intervention of CQR, a decrease in weight gain and an improvement in serum biochemical parameters occurred. In addition, GC-MS detection based urinary metabolomics was applied to characterize the endogenous metabolomic profiling in HFD group and CQR intervened groups. The results indicated that HFD caused the perturbances in the urine metabolomic profiling of rats, and CQR reversed some of these metabolic changes, which was correlated with the dose of CQR. The key differential metabolites were mainly involved in amino acid metabolism, galactose metabolism, pantothenate and CoA biosynthesis and citric acid cycle, pyruvate metabolism and lysine degradation.

### Amino Acid Metabolism

#### Glycine, Serine and Threonine Metabolism

Both glycine and serine are non-essential amino acids that can be converted to each other under certain conditions and can be used for the biosynthesis of glutathione, phospholipids, creatinine and serine (Locasale, 2013). Glycine shows active efficacies in anti-oxidative reactions, metabolic regulation, and neurological protection (Wang et al., 2013). The previous study showed that glycine was negatively related to obesity and regulated lipid metabolism and cholesterol transport (Ding et al., 2016). L-serine is the main carbonyl group for the synthesis of purine nucleotides and deoxy thymidine phosphate. In recent years, L-serine is not only essential for cell proliferation, but also essential for specific functions in the central nervous system (Hirabayashi and Furuya, 2008). The results of this study suggested that glycine, serine, and threonine metabolism might be one of the most affected urinary metabolic pathways. Glycine and serine were significantly lower in the M group than in the N group, and the fold change value of the serine increased in a dose-dependent manner. Therefore, glycine and serine might be potential biomarkers in obese rats.

#### Alanine, Aspartate, and Glutamate Metabolism

Alanine is a critical indispensable substrate for hepatic gluconeogenesis and one of the major amino acids released by skeletal muscle. Glutamine, generally stored in skeletal muscle, is a precursor that organism provides essential nitrogen source for the organism and supplies energy for the synthesis of nucleic acids in the immune system. In addition, glutamine is responsible for improving glucose metabolism and prevent obesity caused by HFD (Sickmann et al., 2010). Alanine, aspartate, and glutamate metabolism was also an influential metabolic pathway in this study. Compared with the N group, L-alanine and glutamate were significantly lowered in the M group. Thus, the reduction of L-alanine and glutamate might be the cause of obesity.

### Arginine and Proline Metabolism

The pathway of arginine and proline metabolism might have great impact on the development of obesity. Glutamine and L-proline were significantly decreased in M group compared with N group. Glutamine might be a crucial potential biomarker in obese model rats for the basis that it not only participates in the metabolism of alanine, aspartate, and glutamate metabolism, but also promotes the process of arginine and proline metabolism. L-proline plays an important role in energy regulation and it can be converted into pyruvate. A possible signaling pathway for L-arginine to play a beneficial role may be the L-arginine-nitrogen oxide pathway. Through this pathway, L-arginine can activate cell signaling proteins, restore insulin sensitivity, regulate glucose homeostasis, promote lipolysis and maintain hormone levels (Arrieta-Cruz and Gutiérrez-Juárez, 2016), thereby producing a weight-loss effect. Further, high-fat diets could reduce proline levels. The results of this experimental study showed that the proline levels in the urine of rats were lowered in M group, which may also be a potential metabolite leading to obesity, and the fold change of proline increased in a dose-dependent manner.

### Galactose Metabolism

D-galactose employs a significant role in the metabolism of galactose. As a down-regulated sugar, galactose can be converted into hydrogen peroxide and aldose by the action of galactose oxidase, thereby forming oxygen radicals and superoxide anions (Lu et al., 2007). Galactose, an important source of energy and key structural elements in complex molecules, is a potential substrate for lactose synthesis and can promote fat mobilization and oxidation (Mohammad et al., 2011). The results of this experiment showed that the content of D-galactose in high-fat diet rats was lower than that in normal rats, and the content of D-galactose could be increased after high-dose and middle-dose administration. Therefore, the decrease of D-galactose means less lipogenesis in high-fat diet rats, which might be notable promising target for CQR to improve obesity.

### Pantothenate and CoA Biosynthesis and Citric Acid Cycle

The citric acid cycle is the main source of ATP production in organisms. Acetyl-coenzyme A (CoA) is an intermediate metabolite in the TCA cycle and plays an important role in energy metabolism. Pantothenic acid is an integral part of acetyl-CoA. It has been reported that the urinary level of pantothenic acid and citrate were significantly increased in rats fed with HFD (Xu et al., 2013; Takahashi et al., 2015). Citrate acts as an intermediate of the citric acid cycle and acts as a signaling molecule to reveal the relationship between energy homeostasis or metabolic state and hemodynamic regulation. The concentration of citrate is closely related to the molecular pathology of the disease, such as hypertension, atherosclerosis and diabetes (He et al., 2004). Therefore, changes in citrate levels in HFD rats may be a signaling response to the disease state. In addition, studies have reported a significant increase of L-valine level in obese or diabetic patients (Mihalik et al., 2010, 2012).

L-proline and pantothenic acid are involved in pantothenate and coenzyme A (CoA) biosynthesis, while citrate is involved in the citric acid cycle and is significantly higher in the M group than in the N group. The HD group inhibited the level of pantothenic acid and citrate, as well as MD and LD groups inhibited the level of TCA activity, which reduces intracellular ATP/AMP levels. The ratio ATP/AMP, which promotes AMPK activation, leads to upregulation of the oxidation of certain fatty acids, lipolysis of lipids and phospholipids, and catabolism of amino acids and glucose, thus resulting in weight loss (Zhou et al., 2001).

### Pyruvate Metabolism and Lysine Degradation

Acetic acid participates in the metabolism of pyruvate, while glycine participates in the lysine degradation, and was significantly lower in the M group than in the N group. It has been reported that acetic acid helps prevent lipid accumulation in the liver. Postprandial blood glucose and insulin levels are negatively correlated with the acetic acid level, and the satiety level. Acetic acid could improve glucose tolerance by reducing gene expression involved in adipogenesis and enhancing fatty acid oxidation in the liver (Petsiou et al., 2014). Glycine is not only involved in the glycine, serine and threonine metabolism, but also in the lysine degradation, which is negatively correlated with obesity. In the meantime, glycine is also involved in the enterohepatic cycle of bile acids (BAs), which are necessary for the lipid absorption and cholesterol homeostasis regulation. With the biodegradation of glycine, lipogenesis is suppressed. Therefore, acetic acid and glycine play a significant role in the body’s metabolism, and their changes are closely related to obesity.

## Conclusion

Obesity, as a chronic metabolic disease, is a serious problem throughout the world. The increased incidence of obesity imposes heavy financial pressure and health burden for the whole society. In present study, the results of body weight, histological analysis, and serum biochemical parameters showed that obesity was improved after treatment with three different doses of CQR. Urinary metabolomics studies based on GC-MS analysis elucidated the perturbances of the HFD on the metabolomic profiling, and the intervene effects of three different doses of CQR. It was found that a total of 22 differential metabolites in HFD group compared with normal group, which were involved in the metabolic pathways of amino acid metabolism, galactose metabolism, pantothenate and CoA biosynthesis, citric acid cycle, pyruvate metabolism and lysine degradation rats. CQR could reverse the changes of some of the differential metabolites, and its effects on metabolomic profiling were in consistent with its dose and anti-obesity phenotype results. These potential metabolites and the pathways in which they participate may become valuable clues or the focus of future research on the anti-obesity mechanism of CQR.

## Data Availability

The original contributions presented in the study are included in the article/Supplementary Material, further inquiries can be directed to the corresponding author.
