# Supplementary material for: Dose-Related Urinary Metabolic Alterations of a Combination of Quercetin and Resveratrol-Treated High-Fat Diet Fed Rats
Source: Front Pharmacol. 2021 Apr 16;12:655563. doi: 10.3389/fphar.2021.655563 (PMC8085560; doi:10.3389/fphar.2021.655563)
Supplement: Supplementary file 1 [file datasheet1.docx]

**Table S1 List of Significant Biomarkers Related to Obesity.**

| **Metabolites** | **M *vs.* N** | | **HD *vs*. M** | | **MD *vs.* M** | | **LD *vs.*M** | |
| --- | --- | --- | --- | --- | --- | --- | --- | --- |
|  | **FC^a^** | **T-test (*p*)** | **FC** | **T-test (*p*)** | **FC** | **T-test (*p*)** | **FC** | **T-test (*p*)** |
| Glutamine | 0.014 | < 0.001 | － | － | 35.59 | 0.046 | 17.11 | 0.010 |
| L-Alanine | 0.028 | < 0.001 | 47.83 | < 0.001 | － | － | 25.32 | < 0.001 |
| L-Proline | 0.063 | < 0.001 | 22.39 | < 0.001 | 12.03 | 0.012 | 6.32 | < 0.001 |
| trans-Aconitic acid | 0.068 | < 0.001 | 12.20 | 0.0030 | － | － | － | － |
| Uric acid | 0.010 | <0.001 | 71.25 | < 0.001 | － | － | － | － |
| Acetic acid | 0.13 | <0.001 | 5.50 | < 0.001 | － | － | － | － |
| 2-Piperidinecarboxylic acid | 0.015 | <0.001 | 72.43 | < 0.001 | 17.32 | 0.012 | 8.49 | < 0.001 |
| Hippuric acid | 0.064 | <0.001 | － | － | 3.77 | 0.017 | － | － |
| Propanoic acid | 0.18 | <0.001 | 3.02 | 0.0048 | 3.05 | < 0.001 | － | － |
| Glycine | 0.22 | <0.001 | 2.57 | 0.0033 | 2.12 | 0.028 | － | － |
| Butanedioic acid | 0.082 | 0.0014 | 4.93 | <0.001 | 5.30 | 0.020 | 3.86 | < 0.001 |
| Butanoic acid | 0.22 | <0.001 | 3.74 | 0.0018 | 3.92 | 0.0045 | － | － |
| Quinolinic acid | 0.30 | <0.001 | 2.91 | <0.001 | 2.84 | 0.0035 | － | － |
| Propanedioic acid | 0.21 | 0.0034 | 6.93 | 0.0014 | 4.91 | 0.0018 | － | － |
| Benzeneacetic acid | 0.15 | 0.0070 | 3.10 | 0.0027 | － | － | － | － |
| D-Galactose | 0.33 | 0.0039 | 3.75 | <0.001 | 3.37 | 0.020 | － | － |
| Hexadecanoic acid | 0.61 | 0.0021 | － | － | － | － | － | － |
| L-Isoleucine | 0.070 | 0.0072 | 6.30 | 0.0039 | 8.44 | 0.0086 | － | － |
| L-Serine | 0.068 | 0.0082 | 30.43 | <0.001 | 9.27 | 0.011 | 7.94 | 0.013 |
| Citric acid | 9.55 | < 0.001 | 0.51 | 0.0020 | 0.57 | 0.013 | 0.64 | < 0.001 |
| L-Valine | 3.87 | <0.001 | － | － | 0.68 | 0.026 | 0.23 | < 0.001 |
| Pantothenic acid | 2.30 | 0.015 | 0.37 | 0.0025 | － | － | － | － |

^a^ Values greater than 1indicate higher levels in M group relative to N group; values less than 1.0 indicate higher levels in N group relative to M group, this comparison method was also applicable in other groups.The short dash line (-) indicates no significant variation.

**Table S2 Relative Metabolic Pathways of Urine Metabolites.**

| **Metabolic pathways** | **Metabolites** | **M *vs.* N** | **HD *vs*. M** | **MD *vs.* M** | **LD *vs.* M** |
| --- | --- | --- | --- | --- | --- |
| Glycine, serine and threonine metabolism | Glycine | ↓ | ↑ | ↑ | － |
| Glycine, serine and threonine metabolism | L-Serine | ↓ | ↑ | ↑ | ↑ |
| Alanine, aspartate and glutamate metabolism | L-Alanine | ↓ | ↑ | － | ↑ |
| Alanine, aspartate and glutamate metabolism | Glutamine | ↓ | － | ↑ | ↑ |
| Galactose metabolism | D-Galactose | ↓ | ↑ | ↑ | － |
| Pantothenate and CoA biosynthesis | Pantothenic acid | ↑ | ↓ | － | － |
| Pantothenate and CoA biosynthesis | L-Valine | ↑ | － | ↓ | ↓ |
| Arginine and proline metabolism | Glutamine | ↓ | － | ↑ | ↑ |
| Arginine and proline metabolism | L-Proline | ↓ | ↑ | ↑ | ↑ |
| Pyruvate metabolism | Acetic acid | ↓ | ↑ | － | － |
| Lysine degradation | Pentanedioic acid | ↓ | ↑ | ↑ | ↑ |
| Lysine degradation | Glycine | ↓ | ↑ | ↑ | － |
| Citrate cycle (TCA cycle) | Citric acid | ↑ | ↓ | ↓ | ↓ |

These metabolites are verified by reference compounds available. The short dash line (–) indicates no significant variation.The arrow denotes the VIP value greater than 1.0 and the up- (or down-) regulation of the arrow represents the relative increased (or decreased) concentration.
